# Biomarker Identification and Pathway Analysis by Serum Metabolomics of Lung Cancer

**DOI:** 10.1155/2015/183624

**Published:** 2015-04-16

**Authors:** Yingrong Chen, Zhihong Ma, Lishan Min, Hongwei Li, Bin Wang, Jing Zhong, Licheng Dai

**Affiliations:** ^1^Huzhou Key Laboratory of Molecular Medicine, Huzhou Central Hospital, Huzhou, Zhejiang 313000, China; ^2^Cardiothoracic Surgery, Huzhou Central Hospital, Huzhou, Zhejiang 313000, China; ^3^Respiratory Medicine, Huzhou Central Hospital, Huzhou, Zhejiang 313000, China; ^4^School of Laboratory Medicine and Life Science, Wenzhou Medical University, Wenzhou, Zhejiang 325035, China

## Abstract

Lung cancer is one of the most common causes of cancer death, for which no validated tumor biomarker is sufficiently accurate to be useful for diagnosis. Additionally, the metabolic alterations associated with the disease are unclear. In this study, we investigated the construction, interaction, and pathways of potential lung cancer biomarkers using metabolomics pathway analysis based on the Kyoto Encyclopedia of Genes and Genomes database and the Human Metabolome Database to identify the top altered pathways for analysis and visualization. We constructed a diagnostic model using potential serum biomarkers from patients with lung cancer. We assessed their specificity and sensitivity according to the area under the curve of the receiver operator characteristic (ROC) curves, which could be used to distinguish patients with lung cancer from normal subjects. The pathway analysis indicated that sphingolipid metabolism was the top altered pathway in lung cancer. ROC curve analysis indicated that glycerophospho-N-arachidonoyl ethanolamine (GpAEA) and sphingosine were potential sensitive and specific biomarkers for lung cancer diagnosis and prognosis. Compared with the traditional lung cancer diagnostic biomarkers carcinoembryonic antigen and cytokeratin 19 fragment, GpAEA and sphingosine were as good or more appropriate for detecting lung cancer. We report our identification of potential metabolic diagnostic and prognostic biomarkers of lung cancer and clarify the metabolic alterations in lung cancer.

## 1. Introduction

Lung cancer is one of the most common cancers worldwide; the prognosis for many patients with lung cancer remains poor. The high mortality and poor prognosis of lung cancer are mainly due to the difficulty of early diagnosis. If patients were diagnosed early, the average 5-year survival rate could be as high as 85% [1]. The development of molecular biology has enabled tumor markers to become a common means of diagnosing cancer. The most widely used lung cancer biomarkers are carcinoembryonic antigen (CEA), cancer antigen 125, cytokeratin 19 fragment (CYFRA21-1), and neuron-specific enolase [2]. However, no validated tumor marker is sufficiently accurate to be useful for diagnosis to date. Therefore, searching for novel diagnostic biomarkers of lung cancer remains difficult.

Metabolomics is a powerful quantitative measurement of low-molecular weight metabolites of an organism at a specified time in specific environmental conditions. Fundamental analytical techniques are used to probe the chemical fingerprint of samples and are an effective tool for screening biomarkers [3, 4], diagnosis [5, 6], and biological pathway characterization [7], specifically and accurately correlating a particular pathway and hence any biomarkers in that pathway with the disruption. Using a more precise selection process for candidate marker identification, metabolomics increases the likelihood of validation of candidate biomarkers in subsequent prospective validation studies [8–10]. The approach also enhances the ability of researchers to use the metabolomic data collected from the biomarker discovery phase to gain insight into disease biology.

Metabolomic studies in lung cancer samples have generally employed techniques such as nuclear magnetic resonance [11], high-performance liquid chromatography/mass spectrometry (HPLC/MS and LC/MS/MS) [12], and gas chromatography/MS (GC/MS). HPLC coupled with quadrupole time-of-flight MS (HPLC-Q-TOF/MS) is widely used in metabolomics because it yields accurate qualitative analysis. Given its high sensitivity, peak resolution, and reproducibility, GC/MS is robust metabolomic tool also widely used in metabolite identification and quantification [13].

Previously [14], we used LC-Q-TOF/MS and GC/MS to compare the metabolite profiles of serum from preoperative patients with lung cancer (PRLC), postoperative patients with lung cancer (POLC), and healthy volunteers (controls). We characterized differences in the metabolomic profiles of the three groups using multivariate statistical analyses: principal components analysis (PCA) and partial least squares discriminant analysis (PLS-DA). From the pattern recognition results, we identified ten potential metabolic biomarkers for lung cancer diagnosis.

In this study, we analyzed the construction, interaction, and pathways of potential lung cancer biomarkers using metabolomics pathway analysis (MetPA) based on the Kyoto Encyclopedia of Genes and Genomes (KEGG) database and Human Metabolome Database to identify the top altered pathways for analysis and visualization. We constructed a diagnostic model using potential serum biomarkers from patients with lung cancer. We assessed their classification performance (specificity and sensitivity) using the area under the curve (AUC) of the receiver operator characteristic (ROC) curve, which might be used to distinguish patients with lung cancer from normal subjects.

## 2. Materials and Methods

### 2.1. Subjects

The Huzhou Central Hospital Ethics Committee approved this prospective study; we obtained informed consent from each participant. Serum samples were collected from 30 healthy volunteers without serious medical illness (controls) and from 30 patients with lung cancer without previous history of other cancers at Huzhou Central Hospital from January 2012 to January 2013. Patients and volunteers were matched according to sex and age. Of the 30 patients, 15 had adenocarcinoma, 12 had squamous cell carcinoma, and three had large cell carcinoma. The patients were also staged according to the 1997 World Health Organization tumor-nodes-metastasis (TNM) staging system by Huzhou Central Hospital pathologists: 15 had stage I disease, seven had stage II disease, and eight had stage III disease. All patients had been newly diagnosed and did not receive any form of medical treatment during the sampling period. Preoperative serum was collected before radical correction. Postoperative serum was collected seven days after surgery. Serum was collected from the controls and patients in the morning after fasting. No anticancer agents were administered to the enrolled patients prior to serum collection. Serum samples were collected at the Huzhou Central Hospital Department of Laboratory Medicine; CEA and CYFRA21-1 levels were determined using a Roche COBAS 6000 automated electrochemiluminescence immunoassay analyzer (Roche Diagnostics GmbH; Mannheim, Germany).

### 2.2. LC-Q-TOF/MS

Serum metabolite profiling was performed on an Agilent 1290 Infinity Liquid Chromatography System (Agilent Technologies, Santa Clara, CA, USA) equipped with a 2.1 × 100 mm C18 reverse-phase column with 1.8 *μ*m particle size (Waters Corp., Milford, MA, USA). The column was maintained at 40°C; the injected sample volume was 4 *μ*L. Gradient conditions were 0–2 min 5% B, 2–17 min linear gradient from 5 to 95% B, and 17–19 min 95% B. Solvent A was 0.1% formic acid-water; solvent B was 0.1% formic acetonitrile. The flow rate was 400 *μ*L/min. MS experiments were performed on an Agilent 6530 Accurate-Mass Q-TOF/MS (Agilent Technologies) equipped with electrospray ionization source. Data for each ionization technique were acquired in positive ion mode. The measurement conditions were capillary voltage 4.0 kV, cone voltage 35 kV, ion source temperature 100°C, and vaporizer temperature 350°C. Nitrogen was used as the nebulizer gas and delivered at a flow rate of 50 L/h; the desolvation gas (nitrogen) was delivered at a flow rate of 600 L/h. The scan range was *m*/*z* 50–1000.

### 2.3. GC/MS

A 1 *μ*L aliquot of derivatized sample was injected splitless into an Agilent 7890A GC system equipped with a 30.0 m × 0.25 mm i.d. fused-silica capillary column with 0.25-*μ*m HP-5 ms stationary phase (Agilent Technologies). The injector temperature was set at 280°C. Helium was used as the carrier gas at a constant flow rate of 1 mL/min through the column. The initial column temperature was 80°C; after 2 min, the temperature was increased to 320°C at a rate of 10°C/min and was held at 320°C for 6 min. The column effluent was introduced into the ion source of an Agilent 5975C Mass Selective Detector. The MS quadrupole temperature was 150°C; the ion source temperature was 230°C. Masses were acquired at *m*/*z* 50–550.

### 2.4. LC-Q-TOF/MS and GC/MS Data Processing

LC data were acquired and processed using Mass Hunter Qualitative Analysis Software (version B.03.01; Agilent Technologies). The MS analysis system was used to identify metabolites corresponding to those in the METLIN database (http://metlin.scripps.edu).

GC total ion chromatograms and fragmentation patterns were autoacquired using GC/MSD ChemStation software (Agilent Technologies). The mass charge ratios and their abundance were compared with a standard mass chromatogram in the National Institute of Standards and Technology (NIST) mass spectra library using ChemStation, which generated a list of similarities per peak as compared with those in the NIST library.

### 2.5. Multivariate Data Analysis

Data were exported into SIMCA-P+ 11.0 software (Umetrics AB, Umeå, Sweden) for multivariate analysis, that is, PCA and PLS-DA. Data are expressed as the mean ± SD. An independent *t*-test (*p* < 0.05) was used to determine whether the candidate biomarkers obtained from PLS-DA modeling were statistically significant between groups at univariate analysis level.

### 2.6. Construction of Metabolic Pathway and Functional Score Analysis

MetPA was used to analyze the construction, interaction, and pathways of the 10 potential lung cancer biomarkers. The MetPA is based on several databases and aids in identifying the top altered pathways for analysis and visualization. In this study, we based the MetPA on the KEGG database (http://www.genome.jp/kegg/) and the Human Metabolome Database (http://www.hmdb.ca/).

### 2.7. Statistical Analysis

Sample distribution was determined using the Kolmogorov-Smirnov test. Data are expressed as means ± SD. Analysis of variance was used to analyze the significance of differences between the three groups. We constructed a diagnostic model using the potential serum biomarkers alone or CEA and CYFRA21-1 combined and used the linear discrimination analysis method for analysis. We assessed specificity and sensitivity using the AUC of the ROC curves. All data were analyzed using SPSS version 19.0 (SPSS Inc., Armonk, NY, USA); the significance level was set to *p* < 0.05. All *p* values were two-sided.

## 3. Results

### 3.1. Identification of Potential Biomarkers

Pattern recognition results identified 10 potential metabolic biomarkers for diagnosing lung cancer (Tables 1 and 2). The serum levels of the potential biomarkers were significantly different in PRLC patients compared with the controls and/or POLC patients. Sphingosine, phosphorylcholine, glycerophospho-N-arachidonoyl ethanolamine (GpAEA), *γ*-linolenic acid, 9,12-octadecadienoic acid, oleic acid, and serine levels were significantly different in the PRLC patients as compared with those of the controls and POLC patients. Prasterone sulfate, *α*-hydroxyisobutyric acid, and 2,3,4-trihydroxybutyric acid levels were statistically different in PRLC and POLC patients as compared with the controls.

### 3.2. Comparison of CEA and CYFRA21-1 between Control and Lung Cancer Groups

Table 3 lists the clinical characteristics of the control, PRLC, and POLC groups. Serum CEA and CYFRA21-1 levels of the PRLC group were higher than those of the control and POLC groups.

### 3.3. Metabolic Pathway and Function Analysis

**Figure 1:**
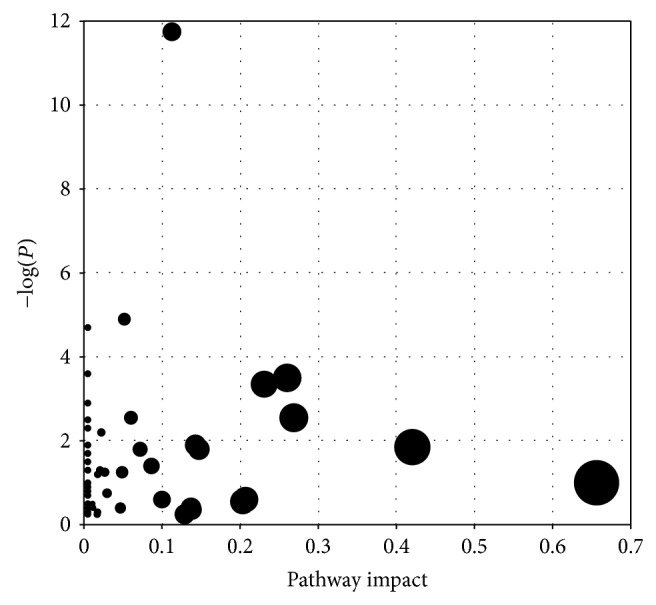
Summary of pathway analysis.

**Figure 2:**
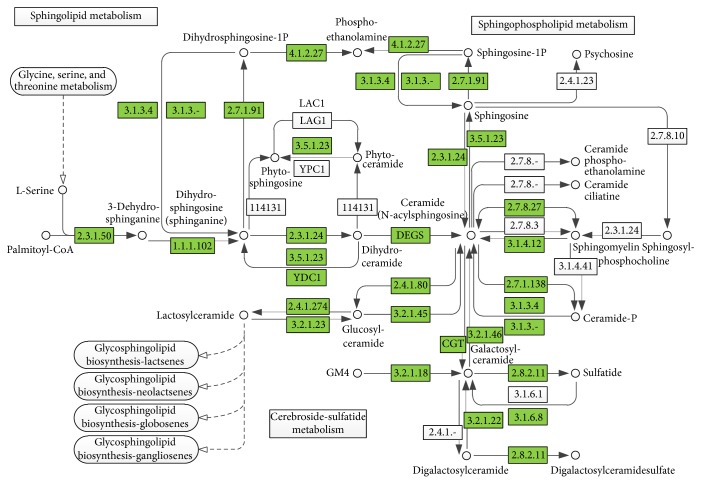
System analysis of metabolomic alterations in lung cancer. The KEGG database was searched for each disrupted metabolite detected; each KEGG pathway was scored according to the pathway impact. The map was generated using the KEGG reference map. Green boxes indicate enzymatic activities with putative analogous cases in humans.

Pattern recognition analysis of metabolites enabled clear separation of the metabolic profiles of the lung cancer groups and the control group [14]. MetPA was used to perform more detailed analysis of the most relevant lung cancer pathways and networks. MetPA of the potential target metabolic pathways revealed that metabolites detected together were important for the host response to lung cancer.  Figure 1 summarizes the pathway analysis, which revealed that the identified metabolites important for lung cancer were mainly responsible for the following metabolism pathways: sphingolipid metabolism, glycine, serine, and threonine metabolism, arginine and proline metabolism, galactose metabolism, and linoleic acid metabolism.  Table 4 lists the detailed results of the pathway analysis.  Figure 2 illustrates the construction of the pathways in detail.

### 3.4. Diagnostic Value of Potential Serum Biomarkers of Lung Cancer

**Figure 3:**
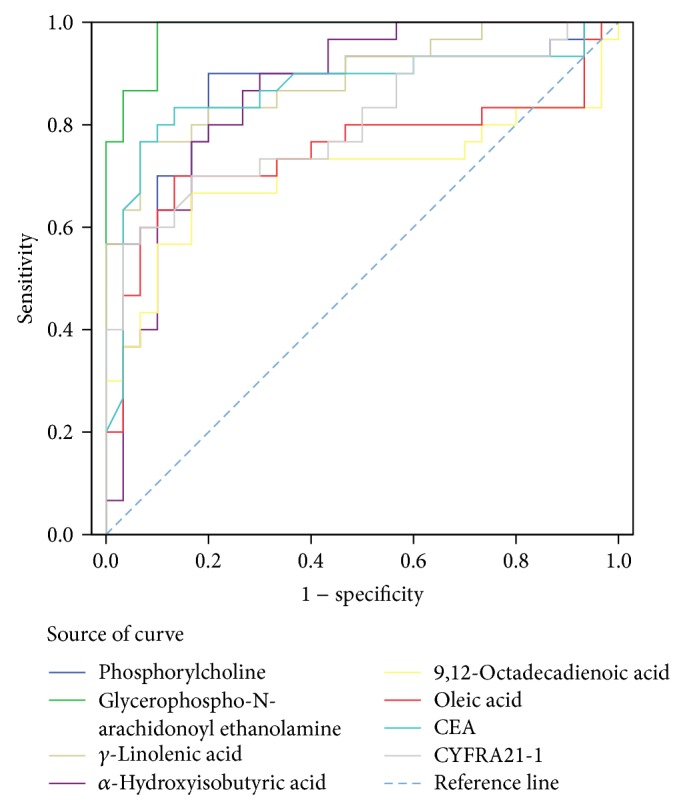
ROC curve analysis of potential serum biomarker and other tumor marker (CEA, CYFRA21-1) levels for differentiating the control group from the PRLC group (high levels of biomarkers in PRLC).

**Figure 4:**
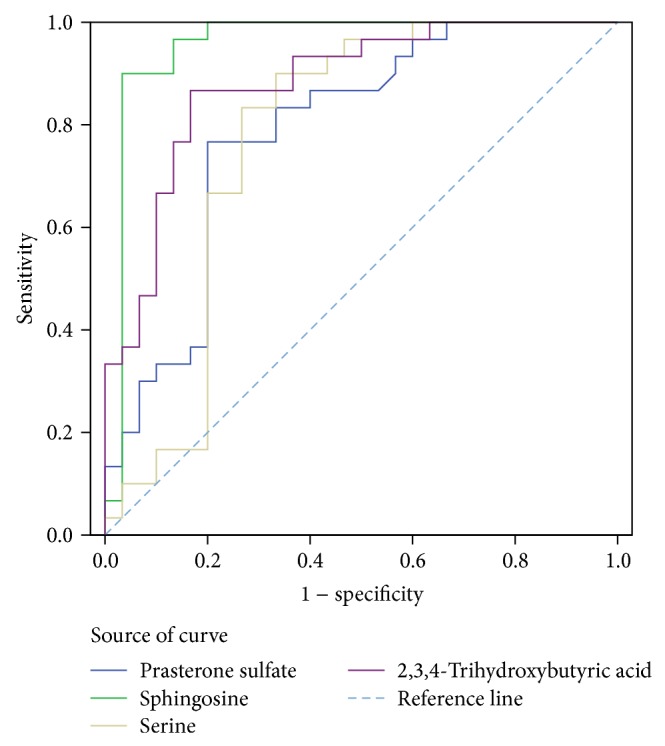
ROC curve analysis of potential serum biomarker and other tumor marker (CEA, CYFRA21-1) levels for differentiating the control group from the PRLC group (low levels of biomarkers in PRLC).

The ROC curve analysis of potential serum biomarker and other tumor marker (CEA and CYFRA21-1) levels for differentiating the control group from the PRLC groups is shown in Figure 3 (high levels of biomarkers in the PRLC group) and Figure 4 (low levels of biomarkers in the PRLC group). The optimal cutoff points as calculated by Youden's index, sensitivities, specificities, and AUC values are listed in Table 5 (high levels of biomarkers in the PRLC group) and Table 6 (low levels of biomarkers in the PRLC group). The AUC value of GpAEA was 0.983 (95% confidence interval [CI] = 0.960–1.000). The optimal cutoff point was 1752.6, indicating 96.67% sensitivity and 90.00% specificity between the control and PRLC groups. The AUC value of sphingosine was 0.957 (95% CI = 0.894–1.000). The optimal cutoff point was 102.76, indicating 90.00% sensitivity and 96.67% specificity between the control and PRLC groups. The GpAEA and sphingosine AUC were significantly greater than those of the other eight potential biomarkers, CEA, or CYFRA21-1 and had similar diagnostic value to that of CEA, allowing differentiation of the PRLC from the control group. These data indicate that GpAEA and sphingosine are high-performance diagnostic biomarkers of lung cancer, where high GpAEA levels and low sphingosine levels indicate lung cancer risk.

**Figure 5:**
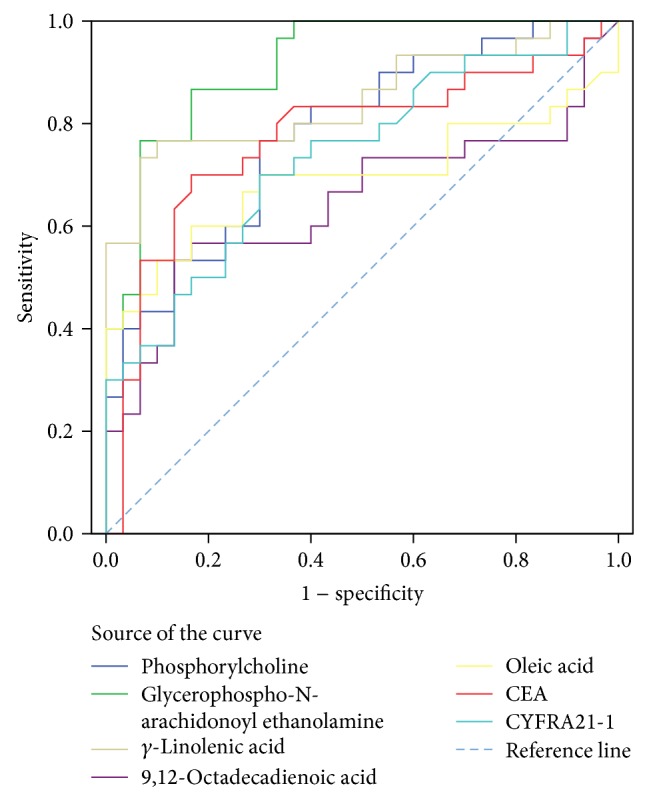
ROC curve analysis of potential serum biomarker levels for differentiating the POLC group from the PRLC group (high levels of biomarkers in PRLC).

**Figure 6:**
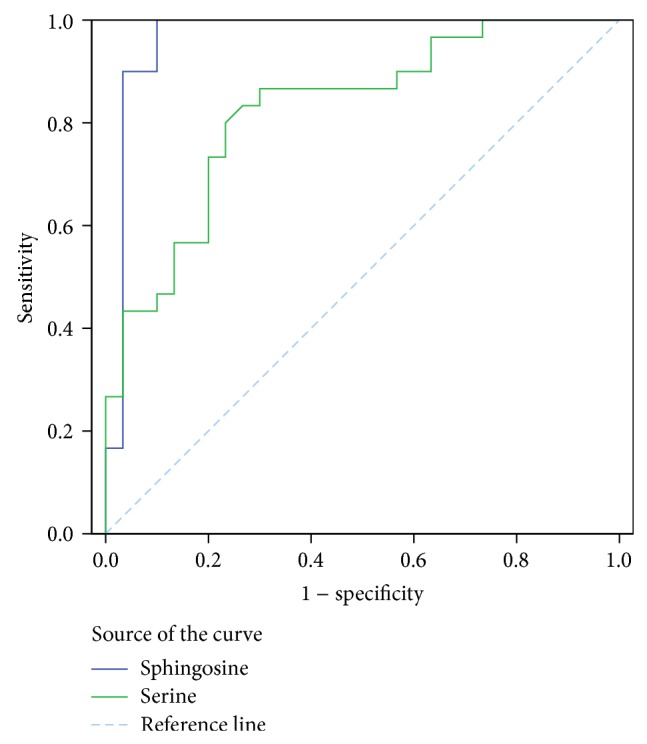
ROC curve analysis of potential serum biomarker levels for differentiating the POLC group from the PRLC group (low levels of biomarkers in PRLC).

ROC curve analysis of the potential serum biomarker levels for differentiating POLC and PRLC patients is shown in Figure 5 (high levels of biomarkers in the PRLC group) and Figure 6 (low levels of biomarkers in the PRLC group). The optimal cutoff points as calculated by Youden's index, sensitivities, specificities, and AUC values are listed in Table 7 (high levels of biomarkers in the PRLC group) and Table 8 (low levels of biomarkers in the PRLC group). The AUC value of GpAEA was 0.916 (95% CI = 0.847–0.984). The optimal cutoff point was 1988.46, indicating 76.67% sensitivity and 93.33% specificity between the POLC and PRLC groups. The AUC value of sphingosine was 0.966 (95% CI = 0.911–11.000). The optimal cutoff point was 86.48, indicating 96.67% sensitivity and 90.00% specificity between the PRLC and POLC groups. The AUC values for GpAEA and sphingosine were significantly greater than those of the other eight potential biomarkers, CEA, or CYFRA21-1 and had similar diagnostic value to that of CEA for differentiating PRLC and POLC patients. These data indicate that GpAEA and sphingosine are high-performance prognostic biomarkers of lung cancer, where high GpAEA levels and low sphingosine levels indicate the risk of lung cancer recurrence.

## 4. Discussion

With its rates of incidence and death being the highest, lung cancer is the most common malignant tumor worldwide. Despite improvements in lung cancer diagnosis and treatment in recent years, the rate of 5-year survival rate remains as low as 16%. Identifying tumor markers can potentially improve lung cancer diagnosis, prognostication, and therapy. Biomarkers are conventionally defined as biological molecules that represent health and disease states. They are typically measured in readily available body fluids, lie outside the causal pathway, and can be used to detect and monitor disease burden and response to treatment [15]. Pathway analysis has been used in metabolomics analysis, vastly extending its clinical relevance and effects [16]. However, the metabolic pathways involved in lung cancer have not been well studied. Emerging techniques in metabolomics have provided a powerful platform for the discovery of novel biomarkers and biochemical pathways that can potentially distinguish diseased and healthy subjects.

In this study, we analyzed the construction, interaction, and pathways of potential lung cancer biomarkers using MetPA based on the KEGG database and the Human Metabolome Database and determined that sphingolipid metabolism was the top altered pathway in lung cancer. ROC curve analysis indicated that GpAEA and sphingosine were potential sensitive and specific diagnostic and prognostic biomarkers of lung cancer. Compared with the traditional diagnostic biomarkers of lung cancer, that is, CEA and CYFRA21-1, GpAEA and sphingosine were as good or more appropriate for detecting lung cancer.

Previous studies have demonstrated that sphingolipids such as sphingosine, ceramide, and sphingosine-1-phosphate are important cell membrane components that play an important role in tumorigenesis [17]. Ceramide is a negative regulator of cell proliferation, inhibiting cell proliferation and promoting apoptosis. Conversely, its metabolite sphingosine-1-phosphate inhibits apoptosis and promotes cell proliferation. Sphingosine, ceramide, and sphingosine-1-phosphate are mutually transformative and maintain homeostasis through enzymatic reactions. Sphingosine kinase, a major rate-limiting enzyme in the cellular synthesis of sphingosine-1-phosphate, regulates both ceramide and sphingosine-1-phosphate by reducing ceramide to generate sphingosine-1-phosphate. Thus, ceramide and sphingosine-1-phosphate homeostasis determines apoptosis and cell proliferation. Inhibiting sphingosine kinase activity increases ceramide and sphingosine levels and decreases sphingosine-1-phosphate levels, inhibiting cell proliferation and promoting apoptosis [18]. Using HPLC-Q-TOF/MS, Yu et al. [19] found that, compared with healthy volunteers, patients with lung cancer had decreased levels of sphingosine. Consistent with this finding, we previously reported that sphingosine levels were significantly decreased in PRLC patients compared with healthy volunteers and POLC patients [5]. In this study, sphingolipid metabolism was the top altered pathway in lung cancer. Sphingosine, ceramide, and sphingosine-1-phosphate are involved in this metabolic pathway, and the decreased sphingosine levels in the PRLC patients may have led to the decreased ceramide levels and increased sphingosine-1-phosphate levels. We believe that the alteration in the levels of these three components in the PRLC patients could have resulted from abnormal activation of the sphingosine kinase pathway, previously implicated in tumor development.

In our study, GpAEA was the other potential sensitive and specific diagnostic and prognostic biomarker of lung cancer. Previous studies have demonstrated that GpAEA could be hydrolyzed by a metal-dependent phosphodiesterase to produce the archetypal endocannabinoid anandamide (AEA), which belongs to the long-chain lipids [20]. A neurotransmitter, AEA, is rapidly hydrolyzed to arachidonic acid and ethanolamine by fatty acid amide hydrolase, which is present very briefly in the nervous system. AEA has two membrane receptors, brain (CB1-R) and spleen (CB2-R) [21], and mediates cellular signal transduction by activating the receptors. On the other hand, AEA affects the physiological function of cells by disrupting cell membrane lipids. After binding CB1-R, AEA activates sphingomyelinase and hydrolyzes sphingomyelin to generate ceramide [22]. In this study, GpAEA levels were increased in PRLC patients compared with that of the controls and POLC patients. We assume that the increased GpAEA levels lead to decreased AEA production and, subsequently, decreased ceramide production. Due to sphingosine, ceramide, and sphingosine-1-phosphate homeostasis, the decreased ceramide levels could lead to decreased sphingosine levels, which is consistent with our previous results. In our metabolic pathway analysis, sphingolipid metabolism was the top altered pathway in lung cancer. Combined with the above analysis, we believe that GpAEA and sphingosine may both be involved in sphingolipid metabolism and hope that they can be developed as sensitive and specific diagnostic and prognostic biomarkers of lung cancer, which require confirmation in further functional studies and large-sample validation.

In conclusion, we demonstrate that studying metabolomics is a simple and noninvasive approach and may be used for identifying diagnostic and prognostic biomarkers of lung cancer. However, as the sample size in this study was small, further studies involving larger populations of patients with lung cancer should be performed to confirm our findings. These investigations would provide important information on the potential of GpAEA and sphingosine as noninvasive markers of lung cancer.

## Figures and Tables

**Table 1:** LC-Q-TOF/MS identification of potential serum biomarkers in lung cancer.

Number	Retention time (min)	*m/z *	Metabolites	Relative mass intensity		
				Control group	Preoperative lung cancer group (PRLC)	Postoperative lung cancer group (POLC)
1	9.47	368.1655	Prasterone sulfate	106.80 ± 31.70	71.99 ± 38.72^*^	50.93 ± 22.26^*^
2	11.89	299.2816	Sphingosine	139.60 ± 38.75	53.33 ± 35.95^∗#^	141.78 ± 42.42
3	12.17	169.0481	Phosphorylcholine	72.70 ± 14.16	133.28 ± 75.49^∗#^	82.17 ± 28.31
4	13.06	501.2862	Glycerophospho-N-arachidonoyl ethanolamine	1355.53 ± 282.89	2722.76 ± 769.63^∗#^	1714.79 ± 399.47
5	16.06	278.2241	*γ*-Linolenic acid	500.34 ± 204.80	1245.99 ± 595.41^∗#^	602.06 ± 226.28

^*^Compared with control group, *p* < 0.05; ^#^compared with postoperative lung cancer group, *p* < 0.05.

**Table 2:** GC/MS identification of potential serum biomarkers in lung cancer.

Number	Retention time (min)	*m/z *	Metabolites	Relative mass intensity		
				Control group	Preoperative lung cancer group (PRLC)	Postoperative lung cancer group (POLC)
1	9.21	131.1089	*α*-Hydroxyisobutyric acid	306.84 ± 153.64	556.54 ± 220.00^*^	803.58 ± 329.41^*^
2	12.23	132.1187	Serine	183.48 ± 96.63	114.92 ± 89.30^∗#^	284.16 ± 184.76
3	18.99	292.2003	2,3,4-Trihydroxybutyric acid	71.89 ± 30.60	24.63 ± 24.13^*^	23.02 ± 14.47^*^
4	30.27	122.1582	9,12-Octadecadienoic acid	9.88 ± 5.79	24.90 ± 18.09^∗#^	13.57 ± 9.30
5	30.38	117.0664	Oleic acid	244.99 ± 131.32	605.66 ± 361.44^∗#^	346.58 ± 164.66

^*^Compared with control group, *p* < 0.05; ^#^compared with postoperative lung cancer group, *p* < 0.05.

**Table 3:** Clinical characteristics of subjects at baseline.

Samples	Control group	Preoperative lung cancer group (PRLC)	Postoperative lung cancer group (POLC)
Sample number	30	30	30
Age	60.35 ± 12.48	61.58 ± 10.67	61.58 ± 10.67
Sex (F/M)	19/11	21/9	21/9
CEA (ng/mL)	1.66 ± 0.72	3.29 ± 1.60^∗#^	2.24 ± 1.42
CYFRA21-1 (ng/mL)	1.30 ± 0.46	3.37 ± 2.66^∗#^	1.53 ± 0.72

^*^Compared with control group, *p* < 0.05; ^#^compared with postoperative lung cancer group, *p* < 0.05.

**Table 4:** Pathway analysis results.

	Total	Expected	Hits	Raw *p*	FDR	Impact
Sphingolipid metabolism	25	0.74	3	0.036	0.47	0.66
Glycine, serine, and threonine metabolism	48	1.42	3	0.17	0.78	0.42
Arginine and proline metabolism	77	2.27	5	0.074	0.66	0.27
Galactose metabolism	41	1.21	4	0.031	0.47	0.26
Linoleic acid metabolism	15	0.44	3	0.36	1.00	0.23

**Table 5:** ROC curves of potential serum biomarker levels for differentiating the control group from the PRLC group.

Marker	Cutoff value	Sensitivity (%)	Specificity (%)	AUC	*p* value^*^	95% CI^a^
Phosphorylcholine	78.32	90.00	80.00	0.874	<0.001	0.780–0.969
Glycerophospho-N-arachidonoyl ethanolamine	1752.60	96.67	90.00	0.983	<0.001	0.960–1.007
*γ*-Linolenic acid	805.17	76.67	93.33	0.889	<0.001	0.806–0.972
*α*-Hydroxyisobutyric acid	365.23	80.00	80.00	0.860	<0.001	0.764–0.956
9,12-Octadecadienoic acid	13.56	66.67	83.33	0.704	<0.001	0.562–0.847
Oleic acid	402.22	70.00	86.67	0.749	<0.001	0.614–0.884
CEA	2.54	76.67	93.33	0.867	<0.001	0.765–0.969
CYFRA21-1	2.02	56.67	96.67	0.803	<0.001	0.690–0.916

^*^Asymptotic significance, null hypothesis: true area = 0.5.

^a^95% confidence interval of the difference.

**Table 6:** ROC curves of potential serum biomarker levels for differentiating the control group from the PRLC group.

Marker	Cutoff value	Sensitivity (%)	Specificity (%)	AUC	*p* value^*^	95% CI^a^
Prasterone sulfate	91.07	76.67	80.00	0.787	<0.001	0.670–0.905
Sphingosine	102.76	90.00	96.67	0.957	<0.001	0.894–1.019
Serine	113.21	83.33	73.33	0.774	<0.001	0.645–0.904
2,3,4-Trihydroxybutyric acid	45.87	86.67	83.33	0.880	<0.001	0.794–0.966

^*^Asymptotic significance, null hypothesis: true area = 0.5.

^a^95% confidence interval of the difference.

**Table 7:** ROC curves of potential serum biomarker levels for differentiating the POLC group from the PRLC group.

Marker	Cutoff value	Sensitivity (%)	Specificity (%)	AUC	*p* value^*^	95% CI^a^
Phosphorylcholine	84.69	76.67	70.00	0.781	<0.001	0.666–0.896
Glycerophospho-N-arachidonoyl ethanolamine	1988.46	76.67	93.33	0.916	<0.001	0.847–0.984
*γ*-Linolenic acid	808.24	76.67	90.00	0.847	<0.001	0.744–0.949
9,12-Octadecadienoic acid	21.29	53.33	86.67	0.645	<0.001	0.499–0.791
Oleic acid	489.02	60.00	80.00	0.694	<0.001	0.550–0.838
CEA	2.90	70.00	83.33	0.772	<0.001	0.646–0.898
CYFRA21-1	1.64	70.00	70.00	0.737	<0.001	0.646–0.863

^*^Asymptotic significance, null hypothesis: true area = 0.5.

^a^95% confidence interval of the difference.

**Table 8:** ROC curves of potential serum biomarker levels for differentiating the POLC group from the PRLC group.

Marker	Cutoff value	Sensitivity (%)	Specificity (%)	AUC	*p* value^*^	95% CI^a^
Sphingosine	86.48	96.67	90.00	0.966	<0.001	0.911
Serine	130.97	80.00	76.67	0.825	<0.001	0.721

^*^Asymptotic significance, null hypothesis: true area = 0.5.

^a^95% confidence interval of the difference.
